# Evaluation of Sexist and Prejudiced Attitudes Toward Homosexuality in Spanish Future Teachers: Analysis of Related Variables

**DOI:** 10.3389/fpsyg.2020.572553

**Published:** 2020-09-04

**Authors:** Davinia Heras-Sevilla, Delfín Ortega-Sánchez

**Affiliations:** ^1^Department of Education Sciences, University of Burgos, Burgos, Spain; ^2^Department of Specific Didactics, University of Burgos, Burgos, Spain

**Keywords:** sexism, heterosexism, homosexuality, teacher training, ideology, attitudes

## Abstract

Discrimination and inequality on the basis of gender and sexual diversity remain prevalent in today’s society (Beck et al., 2010; Dispenza et al., 2012; Dugan et al., 2012; Barrientos and Cárdenas, 2013). These situations of exclusion and rejection show the need to train individuals and organizations in the prevention of violence, harassment and inequality (Kattari et al., 2018). Teacher training, both initial and ongoing, is a fundamental axis of action, and consequently, the study of the opinions and beliefs of students and teachers on these issues. This research, therefore, analyses the sexist and prejudiced attitudes toward homosexuality of future teachers in the Spanish educational system. The sample of this research is made up of 452 students in the Degree in Primary Education Teaching and in the Master’s Degree in Compulsory Secondary Education, Upper Secondary Education, Vocational Training and Language Teaching (MUPES) with an average age of 24.74 (*SD* = 6.51). For the collection of information, a questionnaire was used consisting of questions on sociodemographic and cultural aspects, the Inventory of Ambivalent Sexism (ASI) by Glick and Fiske (1996) validated in Spanish by Expósito et al. (1998), and the Scale of Attitudes of Heterosexuals toward Homosexuals (HATH) by Larsen et al. (1980), validated in Spanish by Barrientos and Cárdenas (2010). The main results include the presence of significant differences (*p* < 0.01) in the levels of ambivalent sexism (hostile and benevolent) and in the maintenance of negative attitudes toward homosexuality according to sex and political ideology.

## Introduction

The scientific and social interest in equal rights and opportunities for women and people with sexual and gender diversity is undeniable. The different countries have been adapting their legislation and policies in order to reach greater levels of equity and equality. Although many rights have been won in recent decades, they are still insufficient to address the needs of these people in a comprehensive manner (Platero, 2009). Discrimination and inequality on the grounds of sexual and gender diversity continue to be a reality in today’s society, as denounced by the study, and subsequent report, conducted by the Organización de las Naciones Unidas (2011) and other recent work on trans, gay, lesbian or bisexual (Beck et al., 2010; Dispenza et al., 2012; Dugan et al., 2012; Barrientos and Cárdenas, 2013; Kattari et al., 2018).

The school context is one of the areas where lesbians, gays, bisexuals, transgender, transsexuals, intersexes, queer and other sexual and gender minority people (LGBTIQ+) suffer most from exclusion and violence (Dugan et al., 2012; Martxueta and Etxeberria, 2014). In this sense, Sánchez Sibony et al. (2018), after a systematic review of studies on harassment and stigmatization in schools for reasons of sexual and gender diversity, determine the existence of a specific form of homophobic bullying. These experiences of bullying are associated with health problems such as depression, anxiety, low self-esteem, substance abuse, isolation, and even the risk of suicide (Birkett et al., 2009; Generelo et al., 2012). There is an urgent need to train educational professionals in the inclusion of this student body and in the prevention of these forms of violence and harassment (Kattari et al., 2018).

Similarly, situations of discrimination and asymmetry toward women are frequent. The most extreme manifestation is gender-based violence, constituting a global health problem that is reaching epidemic rates, as warned by the Organización Mundial de la Salud (2013). In Spain, in 2018, more than 160,000 complaints of gender violence were filed. That same year, 48 women were murdered by their partners or former partners. Scientific studies on this subject reveal the relationship between sexist beliefs and the legitimization of violence, sexual coercion, the use of verbal aggression, tolerance of sexual abuse or a tendency to rape (Forbes et al., 2005; Allen et al., 2009; Durán et al., 2010, 2014). Therefore, the main explanatory models of violence against women give importance to this type of attitude (Echeburúa and Fernández-Montalvo, 1998). Inequality also occurs in other spheres, such as the economy and the workplace. Organización Internacional de Trabajadores (2018) reports that women are responsible for 76.2% of unpaid care work worldwide. In our country, for example, the average hourly wage of women is 17% lower than that of men (Conde-Ruiz and Marra de Artíchamo, 2016). All this leads to questioning the system that legitimizes the differential distribution of work and the different forms of violence against women, the so-called sex/gender system (Rubin, 1996).

The study of the attitudes that underpin the sex/gender system is essential for overcoming inequality, both for reasons of gender and for reasons of sexual diversity (Penna Tosso, 2015; Núñez Noriega, 2016). These attitudes are learned and developed throughout life, as a result of the process of socialization and gender pressure, and involve a willingness to engage in certain more or less inclusive or egalitarian behaviors. In this regard, Egan and Perry (2001) highlight the impact on children’s personality, self-esteem and behavior of pressure for sex-typification from family, classmates or the media. As a result of such pressure, children anticipate evaluative reactions from others, and even from themselves, from gender-differentiated socialization schemes.

It should be recalled that Ajzen (1989, p. 245) defined attitude as “an evaluative disposition toward the object.” Therefore, sexist attitudes can be highlighted, insofar as they imply a predisposition toward differential treatment of men and women. These attitudes, called sexism, condition the way people relate and interact, since they determine what is appropriate and proper to be a man or a woman. Moya and Puertas (2004, p. 216) define sexism as “the set of attitudes about the roles and responsibilities considered appropriate for men and women, as well as the beliefs about relationships that members of both categories should have with each other.” Although in practice sexist attitudes introduce this inequality, they do not always do so out of aversion or rejection. Thus, at present, one can distinguish both discrimination and opposition to women, as well as certain paternalistic and indulgent feelings toward them. In both cases, attitudes of undervaluation and prejudice based on sex are being generated and legitimized.

For all these reasons, Glick and Fiske (1996) consider that sexism is ambivalent, since sexist antipathy is mixed with certain positive feelings toward women. In ambivalent sexism there are, therefore, two differentiated and closely related dimensions, hostile sexism and benevolent sexism (Glick and Fiske, 1996, 2001; Expósito et al., 1998; Moya et al., 2006). The first, hostile sexism, is the most classic form of sexism, so it is also known as old-fashioned sexism or traditional sexism. Following the proposal of Glick and Fiske (1996), in the hostile sexism different components intervene: (a) *dominant paternalism*, characterized by the disdain and subordination of women; (b) *competitive gender differentiation*, based on the undervaluation of women’s qualities, mainly for the public sphere; and (c) *heterosexual hostility*, focused on the “sexual power” of women, and the risks this causes in men. The second dimension, benevolent sexism, has a positive affective tone, leading to behaviors considered prosocial or intimacy seeking. In spite of the positive feelings that the preceptor may have, this dimension should be considered sexism, since in it lies the traditional male domination (Glick and Fiske, 1996; Expósito et al., 1998). Benevolent sexism is made up of three basic components: (a) *protective paternalism*, which sustains the vulnerability and weakness of women, and their need for protection; (b) *complementary gender differentiation*, which considers that women possess qualities and characteristics that are different from those of men, being necessary and positive; and (c) *heterosexual intimacy*, according to which, the emotional and sexual fullness of women depends on men.

The attitudes described above imply inequality and discrimination, since they attribute differential and/or complementary capacities and qualities to men and women. Furthermore, given their heteronormative nature, they hide, deny or reject other sexual and gender identities. In any case, in today’s Western society the more traditional and hostile forms of sexism have diminished, with new and subtle forms of sexism taking their place. This makes that, a good part of the citizenship considers reached the equality and denies the existence of discrimination toward women (Expósito et al., 1998; García-Pérez et al., 2011; Martínez and Paterna-Bleda, 2013). This difficulty in identifying and perceiving sexism makes it resistant, and its eradication is more complex. In this sense, García-Pérez et al. (2011, p. 386) use the term “gender blindness” to refer to this inability to perceive inequality and discriminatory practices.

Heterosexism is similar to sexism in that it implies a disposition toward differential treatment based on the belief in the existence of a hierarchy between different sexual orientations. Underlying heterosexism is the belief that all people are heterosexual and that heterosexuality is more desirable than any other sexual choice. Homosexuality appears, at best, as incomplete, accidental, perverse and, at worst, as pathological, criminal, immoral and destructive of civilization (Borrillo, 2001). As with sexism, heterosexism favors the stigmatization, denigration or denial of any non-heterosexual option, legitimizing and justifying situations of discrimination and violence (Borrillo, 2001; Barón et al., 2013). Furthermore, it has been intimately linked to homophobia. This is defined by Penna Tosso and Sánchez Sáinz (2015, p. 84) as “behavioral, cognitive and/or affective hostility toward those who are supposed to desire or have sexual practices with individuals of their own sex.” For these authors, homophobia would also include the rejection of and discrimination against all sexual and gender identities that threaten the dominant hetero-patriarchal system. In this sense, Núñez Noriega (2016) recalls that the identities accepted and promoted by the sex/gender system need homophobia, since it allows them to delimit and maintain their contours and contents. Homophobia, therefore, supports the established social order and the construction of heterosexual man identity. It is not surprising, therefore, that men, especially in school, adopt homophobic behavior to get away from everything that could be associated with femininity (Blaya et al., 2007).

In the field of education, both sexism and heterosexism are verifiable. Androcentric values still persist among teachers (Anguita and Torrego, 2009). Moreover, this group, like the rest of the population, has difficulty detecting situations of inequality or discrimination against women and girls, and consequently, does not identify sexist teaching practices (Del Castillo and Corral, 2011; García-Pérez et al., 2011; Díaz de Greñu et al., 2013; Gómez-Jarabo and Sánchez, 2017). On the other hand, even today, school curricula and materials are masculinized, with a notable absence of woman models and references (Artal, 2009). The few women who are presented in the curriculum are characterized by victimized, masculinized or dubious social roles, reinforcing traditional and/or paternalistic sexist stereotypes (Molet and Bernad, 2015; Ortega-Sánchez and Pagès, 2016, 2018a). This lack of models extends to sexual and gender diversity (Vidiella, 2012). Unfortunately, little progress has been made in schools to overcome these issues (González Pérez, 2017; Cordón et al., 2019), despite the good predisposition toward equality of teachers (Rebollo et al., 2011; Azorín Abellán, 2014; Piedra et al., 2014). This shows the need for teacher training in gender equality, sexual diversity and coeducation, in order to promote a critical view that serves to identify inequality and transform educational practice (Ortega-Sánchez and Pagès, 2018b, 2020). It is worth asking whether the initial training currently offered favors overcoming sexism and heterosexism. Therefore, this research focuses on studying the sexist attitudes and beliefs toward homosexuality of future Primary Education teachers and future teachers of Compulsory Secondary Education, Upper Secondary Education, Vocational Training and Language Teaching. For this purpose, a feminist perspective has been chosen, since, as indicated by Penna Tosso (2015), Penna Tosso and Sánchez Sáinz (2015), and Núñez Noriega (2016), negative attitudes and rejection of homosexuality and other sexual identities are linked to sexism.

## Materials and Methods

As indicated above, the main objective of this research is to analyze the levels of sexism and prejudice against homosexuality in future primary education teachers and future teachers of Compulsory Secondary and Upper Secondary School Education, Vocational Training, Artistic Education and Languages, as well as to determine the possible differences in sustaining these attitudes according to sex or political ideology. To this end, six working hypotheses have been established, taking into account the nature of the attitudes evaluated (sexist or prejudiced toward homosexuality).

• H_1_: Future teachers will still possess considerable levels of sexism, both hostile and benevolent.
• H_2_: Men in the sample will maintain higher levels of sexism than their women colleagues.
• H_3_: Students with more conservative ideological positions will score higher on measures of sexism (hostile and benevolent) than their peers with ideological positions more liberal or left-wing.
• H_4_: Future teachers will have high levels of prejudice against homosexuality.
• H_5_: Men in the sample will have higher levels of prejudice against homosexuality than their women peers.
• H_6_: Students with more conservative or right-wing ideological positions will score higher on measures of prejudice against homosexuals than their more liberal ideological peers.

### Sample

The sample of this research is made up of 452 students of the Degree in Primary Education Teacher and of the Master’s Degree in Compulsory Secondary Education, Upper Secondary Education, Vocational Training and Language Teaching (MUPES) from three Spanish universities: University of Burgos (UBU), University of Murcia (UM) and University of Valladolid (UVa). The sample was of a non-probabilistic type for convenience (Hernández et al., 2010), attending to intentional criteria, fundamentally, the degree of adaptation of the sample to the research objectives. The participants in the study were between 19 and 58 years of age, with a mean age of 24.74 (*SD* = 6.51). With regard to the distribution by sex, the greater presence of females can be highlighted, since they represent 66.59% of the sample. In addition, 0.44% are intersex people. Regarding sexual orientation, most of the participants in the study are heterosexual (89.06%). On the other hand, 4.91% of the sample declared themselves to be homosexual and 4.24% bisexual. Likewise, 1.79% of those surveyed said they were asexual. With regard to the university training of the sample, 45.80% are enrolled in the Primary Education Teacher Degree, 53.98% study the MUPES and only one subject has another teaching qualification. With respect to the universities in which they are studying, nearly half of the participants in the research come from the UVa (45.58%); followed by 30.53% of students from the UBU and 23.89% of students from the UM (Table 1).

**TABLE 1:** Characteristics of the sample.

	**Frequency**	**Percentage**	***M***	***SD***
Sex (*n = 452)*				
Male	149	32.96		
Female	301	66.59		
Intersex	2	0.44		
Sexual orientation (*n = 448)*				
Asexual	8	1.79		
Homosexual	22	4.91		
Heterosexual	399	89.06		
Bisexual	19	4.24		
Degree (*n = 452)*				
Primary School Teacher	207	45.80		
Master in Teachers from Compulsory Secondary Education	244	53.98		
Other*	1	0.22		
University (*n = 452)*				
University of Burgos (UBU)	138	30.53		
University of Murcia (UM)	108	23.89		
University of Valladolid (UVa)	206	45.58		
Number of hours of training received				
NGT (*n = 233)*			21.26	27.65
NTC (*n = 170)*			25.05	45.15

*Others: Psychopedagogy. NGT: Number of hours of training received on gender equality. NTC: Number of hours of training received on pedagogical issues linked to co-education and the educational treatment gender equality in the classroom.

The descriptive analysis of the training received on issues related to gender equality shows that this type of teaching is not widespread. Just over half of the respondents (56.54%) have received training related to these issues at some point (Table 1). Furthermore, the duration or extension of this training varies from less than 1 h to more than 180 h, and this great dispersion can be seen in the measures of central tendency (*M* = 22.01, *SD* = 27.65; *M*_e_ = 10.00 *M*_o_ = 60.00). On the other hand, in the pedagogical training itself (coeducation and approach to gender equality in the classroom), a greater lack of training is discovered. Only 36.67% of the student population surveyed has received this type of training, that is, more than 60% lack specific training to address equality and coeducation in the classroom. With respect to the duration of this pedagogical training, a great variability is again discovered with interventions that oscillate between 1 and 400 h. Again, the central tendency measures reflect this dispersion (*M* = 31.31, *SD* = 27.65; *M*_e_ = 14.50; *M_o_* = 60.00).

### Instrument

A questionnaire made up of two different blocks was used to collect information. The first of these includes questions on sociodemographic, ideological, religious and training aspects, examining, for example, the number of hours of training received on gender equality or on pedagogical issues linked to co-education and the educational treatment gender equality in the classroom.

The second is made up of two standardized scales on attitudes: (a) the Ambivalent Sexism Inventory of (ASI) by Glick and Fiske (1996) validated in Spanish by Expósito et al. (1998), and (b) the Scale of Attitudes of Heterosexuals toward Homosexuals (HATH) by Larsen et al. (1980), translated into Spanish and validated by Barrientos and Cárdenas (2010) in a sample of Chilean university students.

The Spanish version of the ASI (Glick and Fiske, 1996; Expósito et al., 1998) consists of 22 items formulated in the same direction. It is a Likert-type scale with 6 answer options, ranging from 0 (totally disagree) to 5 (totally agree). It evaluates the level of ambivalent sexism of the participants, differentiating its main components: Hostile Sexism (HS) and Benevolent Sexism (BS). Regarding the internal consistency of the scale, Expósito et al. (1998) obtain very good reliability coefficients, both in the ASI (first study α = 0.88/second study α = 0.90), and in the two subscales that make it up: HS (first study α = 0.87/second study α = 0.89) and BS (first study α = 0.84/second study α = 0.86). The reliability of this research results in significantly higher coefficients, indicating high internal consistency. The analysis of these parameters in the whole scale – ASI – shows an excellent internal consistency (α = 0.934; Ω = 0.945). In the case of HS, excellent reliability coefficients are also obtained (α = 0.934; Ω = 0.946). Finally, the BS coefficients indicate that this subscale also has a very good reliability (α = 0.849; Ω = 0.890).

The Spanish version of the HATH (Larsen et al., 1980; Barrientos and Cárdenas, 2010) is a 5-point Likert-type scale (from 1 = total disagreement to 5 = total agreement), which has 20 items and evaluates the presence of negative attitudes toward homosexuals. A high score on the scale indicates greater prejudice toward this social group (Barrientos and Cárdenas, 2010). The HATH in its original version presented an adequate internal consistency with α = 0.86 (Larsen et al., 1980). Barrientos and Cárdenas (2010) obtained a Cronbach’s Alpha of 0.90, which shows the excellent reliability of the scale in Spanish. In the present study, the reliability presented by HATH is similar, being very good (α = 0.891) or excellent (Ω = 0.928).

### Procedure

A non-experimental quantitative research of a transversal and exploratory nature is chosen, with the purpose of knowing a set of variables (scales) through its “initial exploration at a specific time” (Hernández et al., 2010, p. 152). To this end, the instrument described in the previous section is used to collect information. The administration of the questionnaire is carried out in person and collectively in the different classrooms selected from the universities of Burgos, Valladolid and Murcia, recalling the voluntary and anonymous nature of the participation in the study.

After the data collection procedure, the data are computerized and coded in a database. For this task, together with the analysis, the IBM SPSS Statistics 25 statistical package is used. For the study of the size of the effect of the results extracted, the free software G^∗^Power in version 3.1.9.4. is used, in accordance with the recommendations for its use from Cárdenas and Arancibia (2014).

### Data Analysis

Tests for two or more independent samples and correlations have been used primarily. Parametric statistics are used because of its robustness (Marôco, 2009), “even when the distributions under study have a considerable bias and/or flattening” (Barreira, 2008, p. 170). According to the central boundary theorem, “the larger the sample, the greater the probability that the mean distributions of the variables involved will be normally distributed, even if, individually, they do not have a normal distribution. Therefore, increasing the sample size [as done in this study] reduces the effects of variable non-normality, which increases the robustness of the analysis and makes the transformation of these variables less necessary” (Barreira, 2008, p. 171). Therefore, the following tests are applied: the t Student test, the analysis of variance with ANOVA and *post hoc* contrasts using the Bonferroni test, and Pearson’s correlations. In addition, the justification for the specific use of the ANOVA, a natural extension of the Student *t*, lies in the optimum and moderate robustness of the test in the face of non-compliance with the assumptions of normality and homocedasticity, respectively. This robustness has been demonstrated, moreover, by checking the limitation of the impact of non-compliance with the assumption of normality on the type I error rate (Finch, 2005). It should be noted that Bonferroni’s test assumes homocedasticity of variance, which is difficult to meet in all the groupings proposed in the analysis. However, it is applied assuming the assumptions of planned, simple and complex hypotheses, and understanding that its restrictive character to locate differences gives more value to the results.

The measures proposed by Cohen (1988) are used to study the size of the effect of the results obtained. In the case of the Student *t-tests*, Cohen’s *d* has been calculated. Cohen’s *f* has been applied to the unifactorial ANOVAs, and Cohen’s *d* has also been used in the *post hoc* contrasts with Bonferroni, since they are based on Student’s *t.*

## Results

### Ambivalent Sexism: Hostile Sexism and Benevolent Sexism

This study found low levels of ambivalent (*M* = 0.85, *SD* = 0.81), hostile (*M* = 0.93, *SD* = 1.01), and benevolent (*M* = 0.77, *SD* = 0.77) among study participants.

The application of the Student *t-Test* to the ASI scores and its two subscales BS and HS reveals statistically significant differences (*p* = 0.000) according to gender (Table 2). Men obtain higher scores on BS (*M* = 1.06, *SD* = 0.92) than women (*M* = 0.62, *SD* = 0.63). Although statistically significant differences are found (*p* = 0.000), given the dispersion in scores, a moderate effect size is obtained (*d* = 0.59). A similar situation is found in HS, where men obtain higher scores (*M* = 1.35, *SD* = 1.21) than women (*M* = 0.72, *SD* = 0.81). Here again, highly significant differences (*p* = 0.000) and moderate effect size (*d* = 0.66), although slightly higher than BS, are found. In line with the above, similar results are obtained for the whole scale. A comparison of the ASI scores for men (*M* = 1.20, *SD* = 0.98) and women (*M* = 0.67, *SD* = 0.64), reveals significant differences (*p* = 0.000) with a slightly larger, but moderate effect size (*d* = 0.69). The study of the effect size reveals that the differences found are statistically significant, but with moderate potency. This highlights the unstable and generalizable nature of the differences found on the basis of sex in sexist attitudes, showing the possibility of reducing these differences through education, for example.

**TABLE 2:** Comparison of scores in BS, HS, and ASI according to gender.

	***M***	***SD***	***t***	***gl***	***p***	***d***
Benevolent Sexism – BS						
Man (*n* = 149)	1.06	0.92	5.18	217.46	0.000**	0.59
Woman (*n* = 301)	0.62	0.63				
Hostile Sexism – HS						
Man (*n* = 149)	1.35	1.21	5.77	216.04	0.000**	0.66
Woman (*n* = 301)	0.72	0.81				
Ambivalent Sexism – ASI						
Man (*n* = 149)	1.20	0.98	6.00	212.54	0.000**	0.69
Woman (*n* = 301)	0.67	0.64				

**p < 0.01.

The scores obtained in BS, HS, and ASI in different groups have been compared according to the political ideology of the research participants. The results obtained with the ANOVA show that there are significant differences depending on the ideology (Table 3). Significant differences are observed in BS, HS and ASI (*p* < 0.01), with an acceptable effect size in BS (*f* = 0.35) and high in HS (*f* = 0.58) and ASI (*f* = 0.58). In line with the effect size study, the *post hoc* contrast analysis using the Bonferroni test shows differences between the various ideological groups: extreme left (G1), left-wing (G2), center-left (G3), center (G4), center-right (G5), right-wing (G6) and extreme right (G7). As can be seen in Table 4, a positive trend is detected as we go through the ideological range in HS and ASI, that is, hostile and ambivalent sexist attitudes increase as ideological positioning becomes more conservative. In the case of the BS, given its subtle nature, although variations are detected in the sequence, a positive trend is also observed. In this case, the extreme left group holds a slightly higher level of benevolent or paternalistic sexist attitudes than the left group. The same is true between the center-left people and the center people, with the latter having lower levels of this type of sexism.

**TABLE 3:** ANOVA Comparison of scores in BS, HS and ASI according to political ideology.

		**BS**					**HS**					**ASI**				
	***n***	***M***	***SD***	***F***	***p***	***f***	***M***	***SD***	***F***	***p***	***f***	***M***	***SD***	***F***	***p***	***f***
Extreme left	25	0.53	0.50	15.93	0.000**	0.35	0.29	0.29	23.90	0.000**	0.58	0.42	0.34	24.94	0.000**	0.58
Left-wing	136	0.49	0.51				0.47	0.69				0.48	0.53			
Center-left	83	0.82	0.74				0.97	0.89				0.90	0.76			
Center	99	0.77	0.77				1.10	0.94				0.93	0.77			
Center-right	56	1.13	0.76				1.57	1.12				1.35	0.84			
Right-wing	15	1.41	0.86				1.75	1.28				1.59	0.87			
Extreme right	3	3.36	1.86				4.27	0.45				3.81	0.78			

**p < 0.01.

**TABLE 4:** Comparison and *post hoc* contrasts using Bonferroni’s test of BS, HS, and ASI scores according to declared ideology.

				**Bonferroni**											
				**G1**		**G2**		**G3**		**G4**		**G5**		**G6**	
	***n***	***M***	***SD***	***p***	***d***	***p***	***d***	***p***	***d***	***p***	***d***	***p***	***d***	***p***	***d***
BS															
Extreme left (G1)	25	0.53	0.50												
Left-wing (G2)	136	0.49	0.51												
Center-left (G3)	83	0.82	0.74			0.017*	−0.54								
Center (G4)	99	0.77	0.77												
Center-right (G5)	56	1.13	0.76	0.008**	−0.87	0.000**	−1.08			0.038*	−0.47				
Right-wing (G6)	15	1.41	0.86	0.002**	−1.34	0.000**	−1.67	0.049*	−0.78	0.016*	−0.82				
Extreme right (G7)	3	3.36	1.86	0.000**	−2.36	0.000**	−5.18	0.000**	−3.24	0.000**	−3.21	0.000**	−2.71	0.000**	−1.88
HS															
Extreme left (G1)	25	0.29	0.29												
Left-wing (G2)	136	0.47	0.69												
Center-left (G3)	83	0.97	0.89	0.016*	−0.75	0.001**	−0.65								
Center (G4)	99	1.10	0.94	0.001**	−0.95	0.000**	−0.78								
Center-right (G5)	56	1.57	1.12	0.000**	−1.35	0.000**	−1.31	0.002**	−0.61	0.034*	−0.47				
Right-wing (G6)	15	1.75	1.28	0.000**	−1.80	0.000**	−1.67	0.027*	−0.82						
Extreme right (G7)	3	4.27	0.45	0.000**	−13.04	0.000**	−5.53	0.000**	−4.84	0.000**	−3.59	0.000**	−2.45	0.000**	−2.09
ASI															
Extreme left (G1)	25	0.42	0.34												
Left-wing (G2)	136	0.48	0.53												
Center-left (G3)	83	0.90	0.76			0.001**	−0.67								
Center (G4)	99	0.93	0.77	0.020*	−0.72	0.000**	−0.70								
Center-right (G5)	56	1.35	0.84	0.000**	−1.28	0.000**	−1.37	0.004**	−0.57	0.009**	−0.53				
Right-wing (G6)	15	1.59	0.87	0.000**	−1.97	0.000**	−1.95	0.009**	−0.89	0.016*	−0.84				
Extreme right (G7)	3	3.81	0.78	0.000**	−8.65	0.000**	−6.23	0.000**	−3.83	0.000**	−3.74	0.000**	−2.94	0.000**	−2.58

*p < 0.05, **p < 0.01.

As can be seen in Table 4, the comparison of ideological groups in the BS reveals highly significant differences (*p* < 0.01) between people who declare themselves to be extreme left (*M* = 0.53, *SD* = 0.50) and people in the center-right (*M* = 1.13, *SD* = 0.76), right-wing (*M* = 1.41, *SD* = 0.86), and extreme right (*M* = 3.36, *SD* = 1.86). In all cases, with a high effect size (*d* > 0.80): extreme left and center-right (*d* = −0.87), extreme left and right (*d* = −1.34), and extreme left and extreme right (*d* = −2.36).

The scores obtained in BS by the G2 (*M* = 0.49, *SD* = 0.51) are lower than the rest of the groups. Consequently, people in G3 score higher on the scale (*M* = 0.82; *SD* = 0.74), with significant differences between G2 and G3 (*p* = 0.017). In this case, in addition to a lower significance (*p* < 05), a moderate effect size is found (*d* = −0.54). More conclusive are the results obtained in the comparison of G2 with people from G5 (*M* = 1.13; *SD* = 0.76), G6 (*M* = 1.41, *SD* = 0.86) and G7 (*M* = 3.36, *SD* = 1.86), in all three cases statistically significant differences are found (*p* < 0.01) and a high effect size (*d* > 0.80) (Table 4).

The Bonferroni test finds statistically significant differences (*p* = 0.049) in BS between center-left and right people. In this case, although the significance is lower, a high effect size (*d* = −0.78) appears to confirm these differences. In line with these results, G3 people also differ significantly (*p* = 0.000) in sustaining BS from extreme right people, with a very high effect size (*d* = −3.24).

*Post hoc* contrasts locate differences between the G4 and groups with more conservative ideologies: G5 (*M* = 1.13; *SD* = 0.76), G6 (*M* = 1.41, *SD* = 0.86), and G7 (*M* = 3.36, *SD* = 1.86). In the first case, comparison of G4 and G5, the differences are significant (*p* < 0.05) but with an insufficient effect size (*d* = −0.47). However, between G4 and G6, statistical differences are found (*p* < 0.05) with a high effect size (*d* = *−*0.82), which shows the power of these differences. Coincidentally, the comparison of G4 and G7 reveals differences of high size and significance (*p* = 0.000; *d* = −3.21). Finally, and as can be seen in Table 4, people on the extreme right have higher levels of BS, with statistically significant differences with the rest of the groups (*p* = 0.000), with a high effect size in all of them (*d* > 0.80).

With respect to the HS, the situation is similar to that obtained in the BS. The comparison of ideological groups in HS reveals significant differences (*p* = 0.016) between people who declare themselves to be extreme left (*M* = 0.29, *SD* = 0.29), with people in the center-left (*M* = 0.47, *SD* = 0.69), with an adequate effect size (*d* = −0.75). The power of these results is evident. The differences are more pronounced (*p* < 0.01), if we compare the HS manifested by the persons in G1 with that sustained by people included in G4 (*M* = 1.10, *SD* = 0.94), centre-right (*M* = 1.57, *SD* = 1.12), right (*M* = 1.75, *SD* = 1.28), and extreme right (*M* = 4.27, *SD* = 0.45). In all cases, with a high size of the effect (*d* > 0.80), it can be considered that the differences found in HS, between people on the extreme left (G1) and people who are in moderation (center-left or center) or in the ideological right-wing fork (center-right to extreme right) are stable and generalizable.

The scores obtained in the HS by the G2 (*M* = 0.47, *SD* = 0.89) are lower than those found in the groups G3, G4, G5, G6, and G7, appearing highly significant differences in all cases (*p* < 0.01). The effect size study shows a moderate impact in the comparison with G3 (*d* = −0.65) and G4 (*d* = −0.78), as well as a high effect with the rest of the groups (*d* > 0.80).

The same tendency is discovered in the comparison of the HS held by the center-left and the adhesion to this type of sexism by other groups located in the ideological range of the right. In this sense, statistically significant differences are found with center-right people (*p* = 0.001), although with a moderate effect size (*d* = −0.61). More notable are the differences found with people from the right (*p* = 0.027, *d* = −0.82). and extreme right (*p* = 0.000, *d* = −4.04), since they present a high statistical power.

Comparison of the G4 (*M* = 1.10, *SD* = 0.94) with the rest of the groups finds significant differences (*p* = 0.034) with the center-right persons (*M* = 1.57, *SD* = 1.12), but with an insufficient effect size (*d* = −0.47). Statistical differences (*p* = 0.000) are also found between G4 and G7. In this case, extreme right have higher levels of traditional sexism (*M* = 4.27, *SD* = 0.45), with a high effect size (*d* = −0.3.59). As in BS, extreme right have clearly higher levels of HS than the rest of the groups, with statistically significant differences (*p* = 0.000), with a high effect size in all of them (*d* > 0.80).

The ASI comparative study between ideological groups shows statistical differences between the G1 (*M* = 0.42, *SD* = 0.34) and the groups: G4 (*M* = 0.93, *SD* = 0.77), G5 (*M* = 1.35, *SD* = 0.84), G6 (*M* = 1.59, *SD* = 0.87), and G7 (*M* = 3.81, *SD* = 0.78). In the first case, comparison of G1 and G4, minor differences are found (*p* = 0.020) with a reasonable effect size since it is close to 0.80 (*d* = −0.72). In the remaining cases, highly significant differences (*p* = 0.000) and a high effect size (*d* > 0.80) are found. It can therefore be considered that the differences found in the levels of ambivalent sexism between people on the extreme left (G1) and people on the ideological right (from the center-right to the extreme right) are stable and generalizable.

The scores obtained in the ASI by the G2 (*M* = 0.48, *SD* = 0.53), although higher than those of the G1, are lower than the rest of the groups. Consequently, people from the G3 score higher on the scale (*M* = 0.90; *SD* = 0.76), with significant differences appearing between the G2 and G3 (*p* = 0.001), although with a moderate effect size (*d* = −0.67). A similar situation is found between G2 and G4, with statistical differences (*p* = 0.000) and an average effect size (*d* = −0.70). In contrast, the results obtained in the comparison of G2 with people in G5 (*M* = 1.35, *SD* = 0.84), G6 (*M* = 1.59, *SD* = 0.87), and G7 (*M* = 3.81, *SD* = 0.78) are conclusive. In all three cases, statistically significant differences (*p* = 0.000) and a high effect size (*d* > 0.80) are found. In this case, it is also possible to affirm that the differences found in the levels of ambivalent sexism, between people of the left and people who are located in the ideological sphere of the right (from the center-right to the extreme right) are stable and generalizable.

Groups composed of center-left (G3) and center (G4) people sustain lower levels of ambivalent sexism than center-right, right-wing and extreme right people. The differences found in both groups with G5, are highly significant (*p* < 0.01) but with medium effect size (*d* < *−*0.60), that is, they are not extrapolated to the population. On the other hand, the differences of G3 with G6 and G7, are very powerful with high values of significance (*p* = 0.000) and size of the effect (*d* > −0.80). Similar results are found between G4 and G6, although with lower significance (*p* = 0.016) and similar effect size (*d* = −0.84). More potent are the differences found between G4 and G7, given that extreme right maintain high levels of ambivalent sexism, with statistically significant differences found with all groups (*p* = 0.000), with a large effect size in all of them (*d* > 0.80). It can be stated, despite the small number of extreme right people in the sample, that people of this extreme ideology possess high or excessive levels of sexism in all the variants studied: benevolent, hostile or ambivalent.

### Attitudes Toward Homosexuality

In the case of attitudes toward homosexual people, the scores obtained in the HATH show a positive attitude of the sample toward these people (*M* = 1.30, *SD* = 0.40). A comparison of the HATH scores of men (*M* = 1.43, *SD* = 0.57) and women (*M* = 1.23, *SD* = 0.25) using the Student *t-test* reveals statistically significant differences between the two groups (*p* = 0.000), with men obtaining higher scores on the scale, i.e., those with more negative and prejudiced attitudes toward homosexuals. However, the study of the effect size of these differences shows a moderate size (*d* = −0.52) (Table 5).

**TABLE 5:** Comparison of HATH scores by sex.

	***M***	***SD***	***t***	***gl***	***p***	***d***
Man (*n* = 149)	1.43	0.57	4.01	175.63	0.000**	0.52
Woman (*n* = 301)	1.23	0.25				

**p < 0.01.

Given the potential importance of the beliefs and values held by the sample, the HATH scores of different groups are analyzed comparatively according to the ideology of the research participants. To do this, the analysis of variance is performed with ANOVA and *post hoc* contrasts using the Bonferroni test. The results reveal statistically significant differences (*p* = 0.000) depending on political ideology, with a high effect size (*f* = 0.79) (Table 6).

**TABLE 6:** ANOVA Comparison of HATH scores according to political ideology.

	***n***	***M***	***SD***	***F***	***p***	***f***
Extreme left	25	1.18	0.15	76.88	0.000**	0.79
Left-wing	136	1.18	0.20			
Center-left	83	1.28	0.26			
Center	99	1.30	0.26			
Center-right	56	1.43	0.39			
Right-wing	15	1.55	0.58			
Extreme right	3	4.57	0.55			

**p < 0.01.

As can be seen in Table 7, a positive trend is detected as we move through the ideological spectrum, i.e., prejudiced attitudes toward homosexuals increase as ideological positioning becomes more conservative. Therefore, people who declare themselves to be extreme left obtain lower scores in the HATH (*M* = 1.18, *SD* = 0.15) than people in the center-right (*M* = 1.43, *SD* = 0.39), right-wing (*M* = 1.55, *SD* = 0.58), and extreme right (*M* = 4.57, *SD* = 0.55), with statistically significant differences (*p* < 0.01). The study of the effect size of these differences between groups reveals an adequate or high effect size: extreme left and center-right (*d* = −0.74), extreme left and right-wing (*d* = −1.00) and extreme left and extreme right (*d* = −16.15).

**TABLE 7:** Comparison and *post hoc* contrasts using Bonferroni’s test on HATH scores according to declared ideology.

				**Bonferroni**											
				**G1**		**G2**		**G3**		**G4**		**G5**		**G6**	
	***n***	***M***	***SD***	***p***	***d***	***p***	***d***	***p***	***d***	***p***	***d***	***p***	***d***	***p***	***d***
Extreme left (G1)	25	1.18	0.15												
Left-wing (G2)	136	1.18	0.20												
Center-left (G3)	83	1.28	0.26												
Center (G4)	99	1.30	0.26			0.023*	−0.53								
Center-right (G5)	56	1.43	0.39	0.005**	−0.74	0.000**	−0.93								
Right-wing (G6)	15	1.55	0.58	0.002**	−1.00	0.000**	−1.42	0.017*	−0.83	0.030*	−0.79				
Extreme right (G7)	3	4.57	0.55	0.000**	−16.15	0.000**	−16.19	0.000**	−12.16	0.000**	−12.16	0.000**	−7.92	0.000**	−5.24

*p < 0.05, **p < 0.01.

The results found in the comparison of the G2 with the rest of the groups, show statistically significant differences with all the groups that are not in the ideological left range. The scores obtained in the HATH by the G2 (*M* = 1.18, *SD* = 0.20) are lower than those shown by the G4 (*M* = 1.30, *SD* = 0.26), significant differences appearing (*p* = 0.023). In this case, a moderate effect size is found (*d* = −0.53); which shows that these differences cannot be generalized. A similar situation is found in the comparison of G2 with G5 (center-right people). In this case, the scores are higher (*M* = 1.43, *SD* = 0.39), as well as the significance of the differences found and the size of the effect (*p* = 0.000, *d* = −0.93). In line with these results, the comparison of the G2 with the G6 scores (*M* = 1.55, *SD* = 0.58), also finds statistically significant differences (*p* = 0.000) with a high effect size (*d* = −1.42). Finally, the comparison between G2 and G7 (*M* = 4.57, *SD* = 0.55) reveals statistically significant differences (*p* = 0.000) with a high effect size (*d* > 0.80) (Table 7).

The comparative analysis also finds statistically significant differences (*p* < 0.05) between center-left (G3) people (*M* = *1*.28, *SD* = 0.26) and right-wing (G6) and extreme right (G7) people. In the first case, there are differences with less significance (*p* = 0.017) but with high effect size (*d* = −0.83), evidencing the strength of these differences. In the second case, comparison of G3 and G7 reveals highly significant differences (*p* = 0.000) with a strong effect size (*d* = −12.16). Once again, the differences in the HATH scores between the left positions, in this case moderate approaches, and the clearly right positions are reflected.

As in the center-left group, statistically significant differences (*p* < 0.05) were found between people in the center and those on the right-wing or extreme right, with a high effect size in both cases (*d* > 0.80).

Finally, as can also be seen in Table 7, it is the G7 who score high on the HATH (*M* = 4.57, *SD* = 0.55), with differences of more than 3 points with the other groups. Therefore, highly significant differences have been found (*p* = 0.000), not only with the groups of the left or moderate ideological range, but, with the rest of the right groups, with a high size of the effect in all of them (*d* > 0.80).

### Relationship Between Variables

The results obtained show the existence of a positive, although moderate, correlation between the number of hours of training received on gender equality, and the number of hours of training received on pedagogical issues linked to co-education and the educational treatment gender equality in the classroom (*r* = 0.527, *p* = 0.000) (Table 8).

**TABLE 8:** Pearson’s correlations between the variables age, training hours about gender and coeducation, and the HATH, BS, HS, and ASI scales.

	**Age**	**NGT**	**NTC**	**HATH**	**BS**	**HS**	**ASI**
Age	1						
NGT	0.076	1					
NTC	−0.039	0.527**	1				
HATH	−0.040	0.111	−0.117	1			
BS	0.043	0.096	−0.044	0.472**	1		
HS	0.029	−0.001	−0.040	0.506**	0.681**	1	
ASI	0.038	0.044	−0.045	0.535**	0.891**	0.939**	1

**p < 0.01. NGT, number of hours of training received on gender equality. NTC, number of hours of training received on pedagogical issues linked to co-education and the educational treatment gender equality in the classroom.

It also confirms the existence of an acceptable, though moderate, degree of correlation between HATH and different forms of sexism. In this sense, the correlation found between HATH and BS is slightly lower (*r* = 0.472, *p* = 0.000). On the other hand, the results obtained between HATH and HS (*r* = 0.506, *p* = 0.000) and between HATH and ASI (*r* = 0.535, *p* = 0.000), show a greater interdependence between the variables. It can be considered with nuances that prejudiced attitudes toward homosexual people are positively related to classic and ambivalent sexist attitudes.

Similarly, and as might be expected, there are more consistent correlations between the three types of sexism. Thus, between BS and HS there is a positive and consistent correlation (*r* = 0.681, *p* = 0.000), being more powerful between BS and ASI (*r* = 0.891, *p* = 0.000). Finally, the correlation is extreme and almost perfect between HS and ASI (*r* = 0.939, *p* = 0.000), which may suggest the use of the HS subscale alone. In any case, the clear relationship and interconnection of the different types of sexism is evident, as pointed out by Expósito et al. (1998).

## Discussion

Non-sexist and non-judgmental attitudes toward homosexual persons are a key aspect of achieving equality and eradicating violence, as detailed extensively in the introduction. In this sense, the future teachers studied present a very low level of ambivalent sexism, an aspect that should be highlighted as it contrasts with the first of the hypotheses and with previous research carried out with the same instrument in similar populations (Cárdenas et al., 2010; Rodríguez-Otero and Treviño, 2017; Scandurra et al., 2017; Kuchynka et al., 2018; Bochicchio et al., 2019; Carretero and Nolasco, 2019; Cordón et al., 2019). Likewise, other studies conducted in the educational context, with diverse scientific methodologies, reveal both significant and high levels of sexism, as well as difficulties in identifying situations of gender inequality and androcentric teaching practices (Del Castillo and Corral, 2011; García-Pérez et al., 2011; Díaz de Greñu et al., 2013; Gómez-Jarabo and Sánchez, 2017). Various works indicate that the predisposition toward equality and the attitude of teachers is good in general, but difficulties still appear at the relational level or in educational practice itself (Rebollo et al., 2011; Azorín Abellán, 2014; Piedra et al., 2014). These studies show that “gender blindness” is the main obstacle to coeducation, since there is still great difficulty in identifying and overcoming specific situations of inequality (García-Pérez et al., 2011; Piedra et al., 2014).

As noted above, the students surveyed had low levels of ambivalent sexism, with the level of hostile or traditional sexism being slightly higher. In contrast, Carretero and Nolasco (2019), in a sample of 1.308 students of teaching in Castilla-La Mancha (Spain), find higher levels of sexism than those found in the present study. In addition, they discover a greater presence of benevolent sexism. Coinciding with their results, the research carried out in Extremadura (Spain) by Cordón et al. (2019), with 1.296 students of the Teacher Training Degree in Primary Education, also reveals higher rates of ambivalent sexism (hostile and benevolent), with higher scores in benevolent sexism. On the other hand, Scandurra et al. (2017) find higher levels of sexism than those discovered in the present research in a sample of 438 Italian teachers. Similar findings were made by Cárdenas et al. (2010) in a sample of 220 Chilean university students from various degrees such as Psychology, Engineering, Journalism and Economics. In their case, there were higher levels of ambivalent sexism than those found in this study and a higher incidence of benevolent sexism. For its part, research conducted in Mexico by Rodríguez-Otero and Treviño (2017) finds higher levels of hostile sexism than benevolent sexism in students of Social Work. However, the overall levels of sexism are high, that is, it is a typified and stereotyped group, which leads to a greater presence of the old sexism (Expósito et al., 1998; Moya et al., 2006). The present work not only refers to reduced levels of sexism among future teachers, but also suggests a trend toward resistance to hostile sexism and the equation of both components of ambivalent sexism. This trend can also be seen in the study carried out by Jiménez-García-Bóveda et al. (2014) with 945 mental health professionals in Andalusia. In their case, minimum levels of sexism are discovered and the trend of balance between the two forms of sexism: hostile and benevolent is evident.

As in previous research (Cárdenas et al., 2010; Jiménez-García-Bóveda et al., 2014; Rodríguez-Otero and Treviño, 2017; Scandurra et al., 2017; Kuchynka et al., 2018; Carretero and Nolasco, 2019; Cordón et al., 2019), the results obtained in this research show that men have higher levels of ambivalent sexism (hostile and benevolent) than women, confirming the second hypothesis. The sex variable is relevant in sustaining sexist attitudes, with men having the highest levels of hostile and benevolent sexism. In line with this, research that includes aspects related to sexism such as gender ideology, stereotypes or the double sexual role confirms the greater adherence of men to this type of belief (Heras and Lara, 2009; Clow et al., 2014; Piedra et al., 2014).

With regard to the detailed analysis of the incidence of the two components of ambivalent sexism, as expected, the men studied maintain higher levels of classical sexism, coinciding with the findings of previous research (Carretero and Nolasco, 2019; Cordón et al., 2019; Rodríguez-Otero and Treviño, 2017). However, recent work by Jiménez-García-Bóveda et al. (2014), with Andalusian health workers, finds similar, almost identical levels of hostile and benevolent sexism in men, with the latter being slightly higher. Similarly, Cárdenas et al. (2010) find a greater prevalence of benevolent sexism in the students surveyed, although no major differences are observed between the two components. It can be concluded, therefore, that men have a greater predisposition toward classical forms of sexism. In the case of women, the results obtained contrast with most previous research, since they score higher on the hostile component of sexism. Only the work of Rodríguez-Otero and Treviño (2017), carried out with students in Mexico, shows this women tendency toward traditional sexism. In the present study, however, the adherence of future women teachers to both types of sexism is very low and similar. In this sense, Jiménez-García-Bóveda et al. (2014) found identical scores in hostile and benevolent sexism among the professionals studied. In contrast, the rest of the research consulted detects higher levels of benevolent sexism in women and, consequently, lower levels of hostile sexism (Cárdenas et al., 2010; Carretero and Nolasco, 2019; Cordón et al., 2019). It can be considered that women tend to be more critical of the old sexism, as members of the discriminated groups, in this case women, are more likely to explain the facts as a result of such discrimination (Quiles et al., 2003). On the contrary, given the affective and subtle nature of benevolent sexism, it is easier for women not to identify it as discriminatory, and consequently to sustain it to a greater degree. Therefore, as sexism is structurally overcome and gender awareness is generated, the “gender blindness” decreases; the subtler aspects of sexism begin to be perceived. It is not surprising, therefore, that studies revealing very low levels of sexism, in men and women, show almost identical measures in both components: hostile and benevolent.

With regard to political ideology, several studies show that the most conservative ideological approaches are related to prejudiced attitudes toward groups that are considered inferior or subordinate (Cohrs and Ibler, 2009; Rottenbacher, 2010, 2012; Rottenbacher et al., 2011; Scandurra et al., 2017). Accordingly, the results obtained in this study reveal that political ideology, understood as a continuum from the left to the right, is a determining variable in sustaining sexist attitudes (hostile, benevolent and ambivalent), confirming the third hypothesis under study. People with left-wing ideological approaches have more egalitarian and less sexist attitudes than those who position themselves in the right-wing ideological fork. Moreover, there is an upward trend in adherence to sexism in all its dimensions as the ideological approach becomes more conservative; the small extreme right group has disturbing levels of ambivalent, hostile and benevolent sexism. Similar results are found by Rottenbacher (2010) and Rottenbacher et al. (2011) applying a standardized scale of right-wing authoritarianism. Both of these studies find positive correlations between conservatism and sexism in all its dimensions, evidencing the upward trend described above. Furthermore, as in the present research, Scandurra et al. (2017) find highly significant differences with a high effect size between Italian teachers who define themselves as conservative and those who call themselves moderate or progressive. In contrast, the study by Cárdenas et al. (2010), although it finds a higher level of hostile sexism in right-wing people, does not detect the same circumstance in benevolent sexism. It is the people who position themselves in the ideological center who show the greatest adherence to this component of sexism (BS). The results of this research, and of previous work, suggest that conservative or right-wing political ideology is strongly related to sexism and prejudice against women. Some authors even propose predictive models in this sense (Rottenbacher, 2010; Rottenbacher et al., 2011). However, sexism has a marked socio-cultural character and structures, and therefore is present in all classes, spheres and social sectors.

In relation to attitudes toward homosexuals, as indicated by Pérez-Testor et al. (2010), few studies address this issue in teachers, with slightly more frequent research with students from the Faculties of Education: future teachers, social educators, pedagogues and teachers of secondary, high school, professional training or language teaching (Piedra et al., 2013; Penna Tosso and Mateos Casado, 2014; Penna Tosso, 2015; Penna Tosso and Sánchez Sáinz, 2015; Robles-Reina et al., 2017).

As opposed to the fourth hypothesis, the present study reveals a low level of negative attitudes toward homosexuality among the students surveyed. These results coincide with those obtained by Penna Tosso and Sánchez Sáinz (2015) in a sample of 214 students of the Master in Teachers from Compulsory Secondary Education and Bachiller, Vocational Training and Language Teaching, where they found reduced levels of behavioral homophobia and normalization of homophobic violence, as well as low maintenance of cognitive and affective homophobia. Scandurra et al. (2017) found similar, though slightly higher, levels of homophobia in a sample of 438 practicing teachers. These findings, and those found in the present study, contrast with the results obtained in similar research conducted in our context or abroad. In Italy, Baiocco et al. (2019) found higher levels of prejudice toward homosexuality in a sample of 323 teachers and educators in nurseries, kindergartens and primary schools. In Melilla, Robles-Reina et al. (2017) found a high level of prejudice toward homosexual persons in a sample of 170 students in the Infant Education, Primary Education, Social Education and Business Sciences Grades. For their part, Penna Tosso and Mateos Casado (2014) found, in a large sample of Ecuadorian student teachers (*n* = 1729), a greater proportion of future teachers who presented cognitive and affective homophobia, showing negative attitudes toward homosexuality. However, the same study found that these attitudes do not materialize in behavioral homophobia or fear of stigmatization, with moderate or low rates in these subscales. It can therefore be stated that the future teachers studied have lower levels of prejudice toward homosexuals than those found in other research. In this sense, work with other population groups confirms that the attitudes found in this research are appropriate, and even positive. For example, the research carried out with the same scale by Cárdenas and Barrientos (2008), with Chilean university students, where they obtained higher scores than those found in the present study, can be highlighted.

As in previous research, the results obtained in this study show that men hold higher levels of negative attitudes toward homosexuality than women (Cárdenas and Barrientos, 2008; Penna Tosso, 2015; Lopez and Taype-Rondán, 2017; Robles-Reina et al., 2017; Scandurra et al., 2017), confirming the fifth hypothesis under study. The study by Robles-Reina et al. (2017) reveals the existence of gender differences in both the level of prejudice and the social distance between gays and lesbians. Future women teachers and social educators have more favorable attitudes toward homosexuality, and low levels of distancing with LGBT people. On the contrary, men appear to have higher levels of prejudice and social distancing, which may make it difficult to exercise their future profession with this group. Similar conclusions can be drawn from the meta-study carried out by Penna Tosso (2015) with a sample of more than twenty-five investigations with diverse populations. In most of them, the sex variable is a determining factor in the maintenance of prejudices toward homosexuals, with men having higher levels of homophobia, heterosexism or other negative attitudes toward homosexuality. Using the same scale, Cárdenas and Barrientos (2008) also found differences between woman university students and their man colleagues, with the latter obtaining higher scores on the scale. On the other hand, the study of Scandurra et al. (2017), carried out with Italian teachers, discovers higher levels of prejudice toward sexual minorities in men, finding important differences in both homophobia and transphobia measures. In summary, the results of this research support the existence of a greater disposition to negative attitudes toward homosexuality in men, an aspect linked to the construction of hegemonic masculinities and the maintenance of the sex/gender system (Blaya et al., 2007; Penna Tosso and Sánchez Sáinz, 2015; Núñez Noriega, 2016).

In relation to political ideology, as already indicated, the most conservative positions are related to more hostile or prejudiced attitudes toward groups that are considered inferior, minority or that attempt, or are perceived to attempt, the predominant *status quo* (Cohrs and Ibler, 2009; Rottenbacher, 2010, 2012; Rottenbacher et al., 2011). In this research, political ideology has been shown to be a determining variable in sustaining negative attitudes toward homosexuality, as reflected in the sixth and final working hypothesis. People with left-wing ideological views have more tolerant and positive attitudes toward homosexuality than those in the right-wing ideological bracket. In addition, there is a clear upward trend in the level of prejudice against homosexual people, as the ideological approach becomes more conservative. Again, the extreme right group possesses alarming levels of hostility and prejudice. This finding not only highlights the low acceptance of sexual diversity in this group, but reinforces the concern expressed by several authors about extreme right movements (Bartual-Figueras et al., 2018; López, 2018). Other similar works with university students and graduates also find in people with conservative or right-wing ideologies a greater predisposition to prejudice, rejection or limitation of the rights of homosexuals (Smith-Castro and Molina-Delgado, 2011; Rottenbacher, 2012). In this sense, various works carried out with practicing teachers and/or educators discover similar results, with prejudice being greater among the most conservative people (Scandurra et al., 2017; Baiocco et al., 2019). In contrast, the study by Cárdenas and Barrientos (2008) finds a higher level of negative attitudes toward homosexuality among people who declare themselves to be in the center, an aspect that can be related to the form of questioning used (left-wing, center or right-wing), as it does not discriminate against different degrees of conservatism. On the other hand, the meta-study carried out by Penna Tosso (2015) concludes, as it happens in this research, that right-wing ideology is related to a greater sustaining of prejudices toward sexual diversity. It can be affirmed, therefore, that the ideological positioning conditions the type of attitudes that are maintained toward homosexuality and other sexual diversities (Rottenbacher, 2012; Scandurra et al., 2017), being the most conservative ideological approaches those that are related to greater levels of prejudice, since they are usually related to a greater perception of threat (Cohrs and Ibler, 2009).

## Conclusion

Throughout the discussion, the main conclusions of the research have been drawn and the results obtained in similar works have been shown. Given the scarcity of studies on teachers’ attitudes toward homosexuals, this work can be considered novel, since traditionally the analysis of homophobia and heterosexist attitudes has focused on students at school (Pérez-Testor et al., 2010; Penna Tosso and Sánchez Sáinz, 2015).

The present research has evaluated the presence of sexist attitudes and prejudice toward homosexual people in the future teachers. The results obtained are encouraging, as they show low levels of both forms of prejudice, contrasting with previous research (Robles-Reina et al., 2017; Carretero and Nolasco, 2019; Cordón et al., 2019). This fact may be due to an emerging social change, derived from the growing boom of feminist and LGBTIQ + movements, and the March 8th protests. Therefore, in order to advance and deepen knowledge about gender equality and inclusion of sexual diversities in the educational field, we recommend research on teaching expectations, communicative action or teaching practice. In this regard, it may be useful to use the proposal for gender diagnosis made by Rebollo et al. (2011), which has been used in various research (García-Pérez et al., 2011; Piedra et al., 2014).

As for the possible relationship of the variables analyzed, coinciding with previous research (Expósito et al., 1998; Glick and Fiske, 2001; Rottenbacher et al., 2011), a positive correlation is found between the various forms of sexism studied (ambivalent, hostile, and benevolent) and negative attitudes toward homosexuality. It can be stated that these forms of prejudice are strongly linked (Penna Tosso, 2015; Penna Tosso and Sánchez Sáinz, 2015), since they support the sex/gender system. Therefore, from a feminist perspective, as pointed out by Núñez Noriega (2016), it can be considered that negative attitudes and rejection of non-hegemonic identities are ways of concretizing and perpetuating the dominant patriarchal and heteronormative system.

Among the most remarkable results of the present study is the incidence of ideology, understood as a continuum from left to right or from liberalism to conservatism, in the two measures analyzed, ambivalent sexism (hostile and benevolent) and heterosexual attitudes toward homosexuals. A greater predisposition to prejudice is observed as ideological positioning becomes more conservative. Several studies have already shown this trend in hostile or prejudiced attitudes toward minority groups (Cohrs and Ibler, 2009; Rottenbacher, 2010, 2012; Rottenbacher et al., 2011). These findings reinforce the concern expressed by several authors regarding extreme right movements (Bartual-Figueras et al., 2018; López, 2018) and invite further research and elaboration on these issues.

This study has some limitations that should be highlighted. Firstly, it has been based on incidental sampling, which makes it difficult to generalize the results to the national context, despite the adequate sample size. On the other hand, although sexual and gender diversity has been considered in the design and development of the study, it is advisable to broaden the spectrum by identifying, for example, transgender or queer realities. The results found in relation to ideology reveal the need to broaden this type of issues in future research, using scales that measure ideological approaches and conservatism more accurately, such as those used by Rottenbacher (2010) and Rottenbacher et al. (2011). It should be remembered that the way the questions are asked is carried out conditions the replies found. In this sense, the study of previous training in gender equality and coeducation is also a limitation of this research, since it has focused on the number of hours received. It is convenient to study in depth other aspects of the training such as the contents, the knowledge acquired or the study of the providers of such training. Likewise, it is considered necessary to extend and enrich the study, introducing the notion of sexual prejudice since this includes other diversities and sexual minorities (Herek, 2000). Finally, the low levels of sexism may highlight the need to evaluate sexism and the predisposition to gender equality, widely understood, with other instruments such as the one proposed by Rebollo et al. (2011), aimed specifically at the educational community. In any case, this work supports the need to continue advancing research on sexual prejudice, training, attitudes and the inclusion of sexual and gender diversities in the educational environment.

## Data Availability Statement

The raw data supporting the conclusions of this article will be made available by the authors, without undue reservation.

## Ethics Statement

The studies involving human participants were reviewed and approved by Ethics Committee of the University of Burgos. The participants provided their written informed consent to participate in this study.

## Author Contributions

DH-S and DO-S designed the study, contributed in the process of research, defining the methodology, collecting the information in the different Spanish universities, and wrote the manuscript. DH-S carried out the statistical analysis, interpretation, and discussion of the results. DO-S was in charge of the final review. Both authors contributed to the article and approved the submitted version.

## Conflict of Interest

The authors declare that the research was conducted in the absence of any commercial or financial relationships that could be construed as a potential conflict of interest.
